# Cerebrospinal fluid synaptosomal-associated protein 25 is a key player in synaptic degeneration in mild cognitive impairment and Alzheimer’s disease

**DOI:** 10.1186/s13195-018-0407-6

**Published:** 2018-08-16

**Authors:** Hua Zhang, Joseph Therriault, Min Su Kang, Kok Pin Ng, Tharick A. Pascoal, Pedro Rosa-Neto, Serge Gauthier, the Alzheimer’s Disease Neuroimaging Initiative

**Affiliations:** 1 Department of Neurology, the First Affiliated Hospital of Chongqing Medical University, Chongqing, 400016 China; 2 The McGill University Research Centre for Studies in Aging, McGill University, Montreal, Canada; 3 Department of Neurology, National Neuroscience Institute, Singapore, Singapore

**Keywords:** Alzheimer’s disease, Amyloid-β, Synaptic loss, Synaptosomal-associated protein 25

## Abstract

**Background:**

There is accumulating evidence that synaptic loss precedes neuronal loss and correlates best with impaired memory formation in Alzheimer’s disease (AD). Cerebrospinal fluid (CSF) synaptosomal-associated protein 25 (SNAP-25) is a newly discovered marker indicating synaptic damage. We here test CSF SNAP-25 and SNAP-25/amyloid-β42 (Aβ42) ratio as a diagnostic marker for predicting cognitive decline and brain structural change in the Alzheimer’s Disease Neuroimaging Initiative (ADNI) database.

**Methods:**

We stratified 139 participants from the ADNI database into cognitively normal (CN; *n* = 52), stable mild cognitive impairment (sMCI; *n* = 22), progressive MCI (pMCI; *n* = 47), and dementia due to AD (*n* = 18). Spearman correlation was performed to test the relationships between biomarkers. Overall diagnostic accuracy (area under the curve (AUC)) was obtained from receiver operating curve (ROC) analyses. Cox proportional hazard models tested the effect of CSF SNAP-25 and SNAP-25/Aβ42 measures on the conversion from MCI to AD. Relationships between the CSF SNAP-25 levels, SNAP-25/Aβ42 ratio, and diagnostic groups were tested with linear regressions. Linear mixed-effects models and linear regression models were used to evaluate CSF SNAP-25 and SNAP-25/Aβ42 as predictors of AD features, including cognition measured by the Mini-Mental State Examination (MMSE) and brain structure and white matter hyperintensity (WMH) measured by magnetic resonance imaging (MRI).

**Results:**

CSF SNAP-25 and SNAP-25/Aβ42 were increased in patients with pMCI and AD compared with CN, and in pMCI and AD compared with sMCI. Cognitively normal subjects who progressed to MCI or AD during follow-up had increased SNAP-25/Aβ42 ratio compared with nonprogressors. CSF SNAP-25, especially SNAP-25/Aβ42, offers diagnostic utility for pMCI and AD. CSF SNAP-25 and SNAP-25/Aβ42 significantly predicted conversion from MCI to AD. In addition, elevated SNAP-25/Aβ42 ratio was associated with the rate of hippocampal atrophy in pMCI and the rate of change of cognitive impairment in CN over the follow-up period.

**Conclusions:**

These data suggest that both CSF SNAP-25 and SNAP-25/Aβ42 ratio are already increased at the early clinical stage of AD, and indicate the promise of CSF SNAP-25 and SNAP-25/Aβ42 ratio as diagnostic and prognostic biomarkers for the earliest symptomatic stage of AD.

## Background

Alzheimer’s disease (AD) is the most prominent cause of dementia in the elderly. AD is characterized by early loss of synapses in specific brain regions, beginning in the hippocampus and spreading to the neocortex and limbic system, eventually leading to memory impairments [1–5]. The loss of synapses in AD is greater than the loss of neurons in the cortex, indicating that the synaptic damage precedes the loss of neuronal cell bodies [6–9]. Neuropathological studies have revealed that synaptic loss is already evident at the stage of mild cognitive impairment (MCI) of AD [10, 11]. In addition, synaptic loss is closely related with the severity of clinical disease [5]. In view of the above reasons, if there is a biomarker that reflects this pathophysiological process, it may be used to study disease mechanisms, improve tools for early diagnosis, predict progression of disease, and monitor the effects of drugs on reducing the rate of synaptic degeneration in clinical trials of disease-modifying therapies for AD [12]. Therefore, biomarkers that can track synaptic dysfunction in AD may prove useful for more accurate disease staging as well as population enrichment of disease-modifying clinical trials [5].

Synaptic damage can be detected at the earliest stages of AD. MCI patients exhibit loss of presynaptic proteins such as synaptophysin and synaptosomal-associated protein 25 (SNAP-25) and postsynaptic markers such as postsynaptic density-95 and Shank 1 [13]. SNAP-25 is a widely distributed membrane-associated protein that is mainly localized in nerve terminals in the brain. Within the nerve terminals, SNAP-25 is involved in the docking and/or fusion of synaptic vesicles to the plasmalemma, a process essential for synaptic vesicular exocytosis [14]. A study has recently reported increased levels of cerebrospinal fluid (CSF) SNAP-25 in AD patients [15].

However, it is still unknown whether CSF SNAP-25 levels increase at the early clinical stage of AD, and whether CSF SNAP-25 is correlated with other core features of AD such as amyloid-β (Aβ) pathology, structural brain changes, and cognitive decline. In the present study, we tested the hypotheses that CSF SNAP-25 levels and SNAP-25/Aβ42 ratio increase at every stage of AD and improve the diagnostic accuracy for AD compared with other core biomarkers. We also tested the hypotheses that CSF SNAP-25 levels and SNAP-25/Aβ42 ratio have associations with Aβ pathology and changes in AD cognition and brain structure, as measured by the Mini-Mental State Examination (MMSE), Alzheimer’s Disease Assessment Scale cognitive subscale (ADAS-cog), and magnetic resonance imaging (MRI).

## Methods

### Database description

Data used in the preparation of this article were obtained from the Alzheimer’s Disease Neuroimaging Initiative (ADNI) database. The ADNI was launched in 2003 as a public-private partnership, led by Principal Investigator Michael W. Weiner, MD. The primary goal of ADNI has been to test whether serial MRI, positron emission tomography (PET), biological markers, and clinical and neuropsychological assessments can be combined to measure the progression of MCI and early AD. Further information can be found at http://www.adni-info.org.

From the dataset, we selected all participants between 55 years and 90 years (inclusive) of age who had completed lumbar puncture, MMSE, ADAS-cog, Clinical Dementia Rating (CDR) scale, and MRI. Selected individuals were classified as cognitively normal (CN; *n* = 52), stable mild cognitive impairment (sMCI; *n* = 22), progressive MCI (pMCI; *n* = 47), and dementia due to AD (*n* = 18) according to clinical and behavioral measures provided by the ADNI. In a subanalysis, we also describe 19 CN subjects who progressed to MCI (*n* = 12) or AD (*n* = 7) during follow-up.

### Classification criteria

The criteria for CN included an MMSE score ranging between 24 and 30, and a CDR score of 0 [16, 17]. The criteria for MCI included the presence of a subjective memory complaint, with an MMSE score between 24 and 30, a CDR of 0.5, preserved activities of daily living, and an absence of dementia [18]. In addition to the NINCDS/ADRDA criteria for probable AD, AD dementia subjects had MMSE scores between 20 and 26 and a CDR of 0.5 or 1.0 [19]. We defined sMCI as MCI subjects not progressing to AD during at least 2 years of follow-up and pMCI as MCI subjects progressing to AD at any time during follow-up [12]. We excluded subjects who were diagnosed with MCI at baseline but reverted to CN during follow-up, as well as subjects who were diagnosed with AD at baseline but reverted to MCI during follow-up. (Further information about the inclusion/exclusion criteria may be found at www.adni-info.org, accessed February 2018.)

### Standard protocol approvals and patient consents

The ADNI study was approved by the Institutional Review Boards of all the participating institutions. Informed written consent was obtained from all subjects at each center.

### CSF analyses

CSF Aβ42, total-tau (t-tau), and phosphorylated-tau at threonine 181 (p-tau) were measured using the multiplex xMAP Luminex platform (Luminex Corp., Austin, TX, USA) and Innogenetics INNO-BIA AlzBio3 (Innogenetics, Ghent, Belgium) immunoassay reagents as described previously [20]. Subjects were classified as Aβ positive or negative using a previously established CSF Aβ42 cutoff < 192 pg/ml [20]. Mouse anti-human SNAP-25 antibodies were used for the development of an Erenna® immunoassay assay according to an agreement between Singulex, Inc. (Alameda, CA, USA) and Washington University. A sandwich enzyme-linked immunosorbent assay (ELISA) was developed using the Erenna® immunoassay system to measure SNAP-25 in CSF. Prior to the assay, all samples were centrifuged (11,000 g × 3 min) to remove particulates. Then, 100-μL standards or CSF diluted fourfold were combined with 100 μL antibody-coated microparticles diluted in Blocker Casein in TBS plus 1% Tween-20 for measurement of SNAP-25 in CSF. The assay plate was incubated for 2 h on a plate shaker set to 525 rpm. Microparticles were then magnetically separated and washed once using an Agilent (Santa Clara, CA, USA) Bravo Automated Liquid Handling Platform using Singulex wash buffer. Fluorescent dye-labeled detection antibody diluted in Blocker Casein in TBS plus 1% Tween-20 (20 μL per well) was added and incubated for 1 h. After washing the magnetic microparticles five times, 20 μL per well of Singulex elution buffer was added for 10 min to separate the detection antibody from the microparticles. Eluted antibodies were then transferred with the Bravo instrument to a clean 384-well plate for reading in the Erenna® immunoassay system. All of the CSF data used in this study were obtained from the ADNI files “UPENNBIOMK5–8.csv” and “FAGANLAB_07_15_2015.csv” (accessed February 2018). Further details of ADNI methods for CSF acquisition and measurements and quality control procedures can be found at www.adni-info.org.

### Cognitive assessment

To assess the global cognitive performance we used the MMSE and ADAS-cog scores. MMSE and ADAS-cog scores were selected at six time points: baseline, 6 months, 12 months, 18 months, 24 months, 36 months, and 48 months. The data used in this study was obtained from the ADNI files “MMSE.csv” and “ADAS_ADNI1. csv” (accessed February 2018).

### Neuroimaging methods

To investigate neurodegeneration we used the hippocampal and ventricular volumes. The white matter hyperintensity (WMH) volume, a cerebrovascular disease marker, was also obtained. Those data were obtained from the ADNI files “FOXLABBSI_08_04_17.csv”, “UCSDVOL.csv”, and “UCD_ADNI1_WMH.csv” (accessed February 2018). All the imaging data were selected at five time points: baseline, 6 months, 12 months, 24 months, and 36 months. The neuroimaging methods used by ADNI have been described previously [21]. Further details for ADNI image acquisition and processing can be found at www.adni-info.org/methods.

### Statistical methods

Analysis of covariance (ANOVA) and chi-square analyses were performed to test for significant differences between groups on baseline demographics. Associations between the CSF SNAP-25 levels and SNAP-25/Aβ42 ratio and diagnostic groups were tested with multiple-variable linear regression, adjusted for age and gender. To evaluate whether Aβ influenced these associations, we included the interaction between diagnosis and Aβ positivity as a predictor in the model.

Spearman correlation was used to test associations between SNAP-25 and SNAP-25/Aβ42 and other core biomarkers. Overall diagnostic accuracy (the area under the curve (AUC)) was obtained for each biomarker using receiver operating curve (ROC) analyses. The differences between two AUCs derived from all pairs of two different variables were tested using bootstrap methods.

The associations of SNAP25 and SNAP-25/Aβ42 with the incidence of AD were assessed by calculating hazard ratios (HRs) with 95% confidence intervals (CIs) using Cox proportional hazard regression analysis with adjustment for age and sex. SNAP25 and SNAP-25/Aβ42 were categorized into two groups by the median of each biomarker when conducting Cox proportional hazard regression analysis.

For MMSE, ADAS-cog, hippocampal and ventricular volumes, and WMH, intercepts (baseline values) and slopes (rates of change) were derived using linear mixed effects models. The intercept and slopes were then used as outcomes in linear regression models with SNAP-25 and SNAP-25/Aβ42 as predictors (adjusted for age and gender; and for education for MMSE and ADAS-cog; and for intracranial volume for hippocampal and ventricular volumes) within diagnostic groups. All statistics were performed using R (v. 3.4.2) and SPSS version 20. Statistical significance was defined as *p* < 0.05 for all analyses.

## Results

### Demographic results

The demographics and biomarker characteristics of the study subjects are presented in Table 1. There were no differences in age or educational level among the groups. Some group cohorts differed significantly for gender, percentage of individuals with the APOE ε4 genotype, mean MMSE, mean ADAS-cog, biomarker levels, and follow-up time (Table 1).

**Table 1:** Demographics of subjects at baseline

Characteristics	CN (*n* = 52)	sMCI (*n* = 22)	pMCI (*n* = 47)	AD (*n* = 18)
Age (years)	76.2 (5.1)	76.0 (5.1)	73.1 (6.6)	74.3 (7.0)
Gender, female (%)	22 (42.3%)	7 (31.8%)^d^	14 (29.8%)^d^	11 (61.1%)
Education (years)	15.7 (3.3)	16.8 (2.4)	15.9 (2.7)	15.2 (2.9)
APOE ε4, *n* (%)	10 (19.2%)^c,d^	7 (31.8%)^c,d^	28 (59.6%)^a,b^	13 (72.2%)^a,b^
MMSE	29.3 (1.0)^b,c,d^	27.2 (1.4)^a,d^	26.6 (1.6)^a,d^	24.2 (2.1)^a,b,c^
ADAS-cog	5.9 (2.8)^b,c,d^	9.2 (3.8)^a,c,d^	12.5 (4.3)^a,b,d^	18.2 (6.1)^a,b,c^
Aβ42 (pg/ml)	211.4 (56.2)^b,c,d^	181.0 (54.7)^a,c,d^	147.5 (47.8)^a,b^	136.1 (27.3)^a,b^
t-tau (pg/ml)	72.7 (28.5)^c,d^	84.7 (54.0)^d^	107.8 (49.3)^a,d^	153.2 (78.7)^a,b,c^
t-tau/Aβ42	0.399 (0.283)^c,d^	0.553 (0.492)^c,d^	0.820 (0.501)^a,b,d^	1.138 (0.569)^a,b,c^
p-tau (pg/ml)	24.6 (10.4)^c,d^	28.1 (15.7)^c,d^	39.5 (16.5)^a,b^	45.8 (16.4)^a,b^
p-tau/Aβ42	0.135 (0.095)^c,d^	0.184 (0.147) ^c,d^	0.303 (0.162)^a,b^	0.350 (0.145)^a,b^
Follow-up (years)	7.5 (2.6)^b,c,d^	4.8 (2.5)^a,d^	5.7 (2.6)^a,d^	2.6 (0.7)^a,b,c^

Values are shown as mean ± standard deviation unless otherwise indicated

*p* values indicate the values assessed with analyses of variance for each variable except gender and APOE ε4, where a contingency chi-square was performed. Post-hoc analysis provided significant differences between groups: ^a^ versus CN; ^b^ versus sMCI; ^c^ versus pMCI; ^d^ versus AD.

*Aβ* amyloid-β, *AD* Alzheimer’s disease, *ADAS-cog* Alzheimer’s Disease Assessment Scale cognitive subscale, *APOE* apolipoprotein E, *CN* cognitively normal, *MMSE* Mini-Mental State Examination, *pMCI* progressive mild cognitive impairment, *p-tau* phosphorylated tau, *sMCI* stable mild cognitive impairment, *t-tau* total tau

### CSF SNAP-25 levels and SNAP-25/Aβ42 ratio in different diagnostic groups

**Fig. 1:**
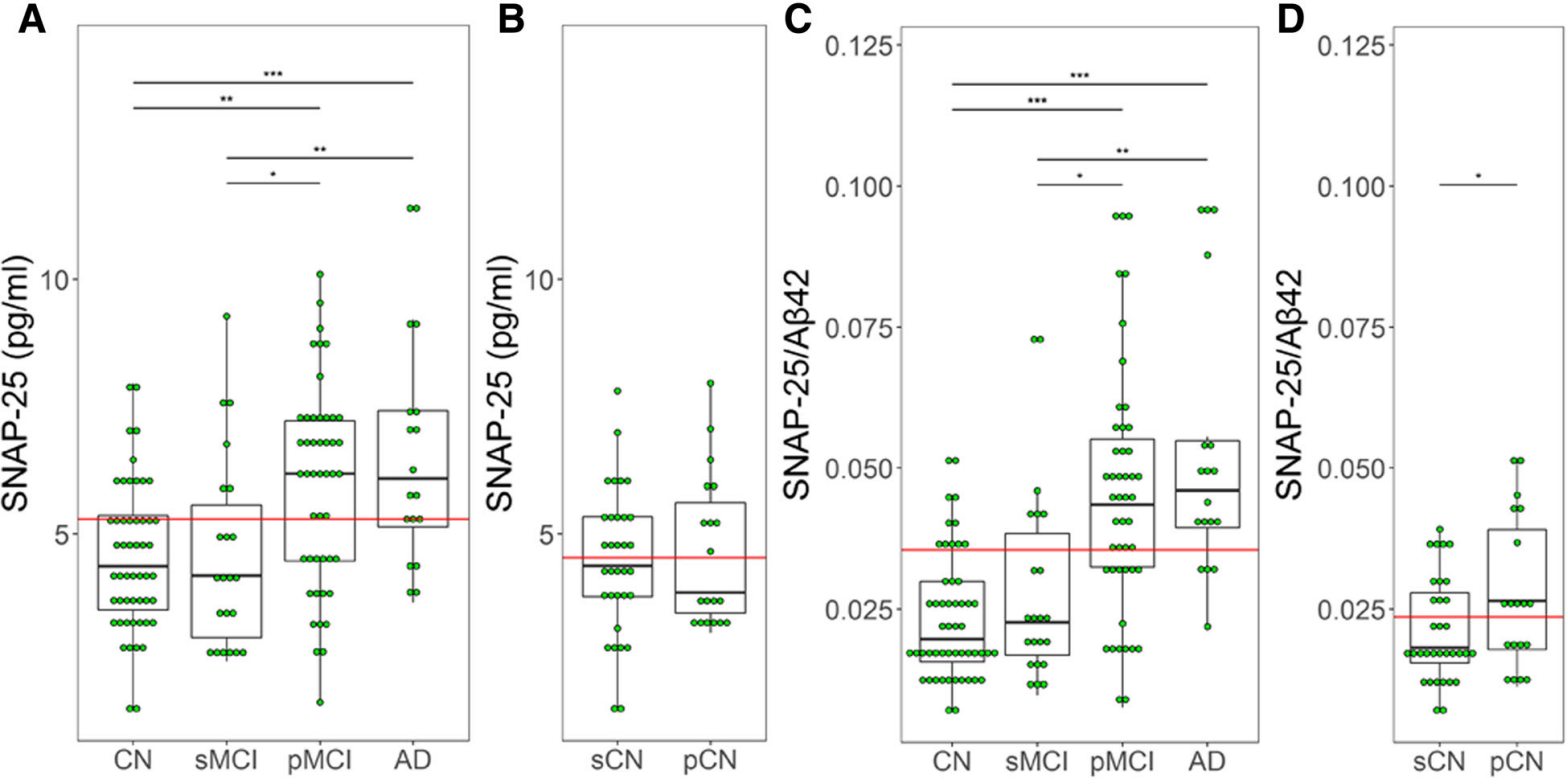
CSF SNAP-25 levels and SNAP-25/Aβ42 ratio in different diagnostic groups. CSF SNAP-25 levels (**a**) and SNAP-25/Aβ42 ratio (**c**) in different diagnostic groups. CSF SNAP-25 levels (**b**) and SNAP-25/Aβ42 ratio (**d**) in the healthy control subjects who progressed to MCI or AD (progressive healthy controls). Differences between groups were tested by multiple-variable linear regression, adjusted for age and sex. *p < 0.05, **p < 0.01, ***p < 0.0001. Aβ amyloid-β, AD Alzheimer’s disease, CN cognitively normal, pCN progressively cognitive normal, pMCI progressive mild cognitive impairment, sCN stably cognitive normal, sMCI stable mild cognitive impairment, SNAP-25 synaptosomal-associated protein 25

CSF SNAP-25 levels were significantly higher in patients with pMCI (*p* < 0.01) and AD (*p* < 0.0001) compared with CN. Higher SNAP-25 levels were also found in both pMCI (*p* = 0.037) and AD (*p* < 0.01) compared with sMCI. However, there were no differences between CN and sMCI, and similarly between pMCI and AD (Fig. 1a). SNAP-25/Aβ42 ratio also showed the same trend in the different diagnostic groups (Fig. 1c). Among the CN, 19 individuals progressed to MCI or AD during follow-up (progressively cognitive normal (pCN)). These participants had significantly higher SNAP-25/Aβ42 ratio (*p* = 0.039) compared with stably cognitive normal (sCN) individuals who did not progress to MCI or AD (Fig. 1d). However, The Aβ42 levels for pCN are less than that of sCN (*p* = 0.042, data not shown). SNAP-25 levels were similar between sCN and pCN (Fig. 1b).

### Associations between CSF SNAP-25 and Aβ pathology

**Fig. 2:**
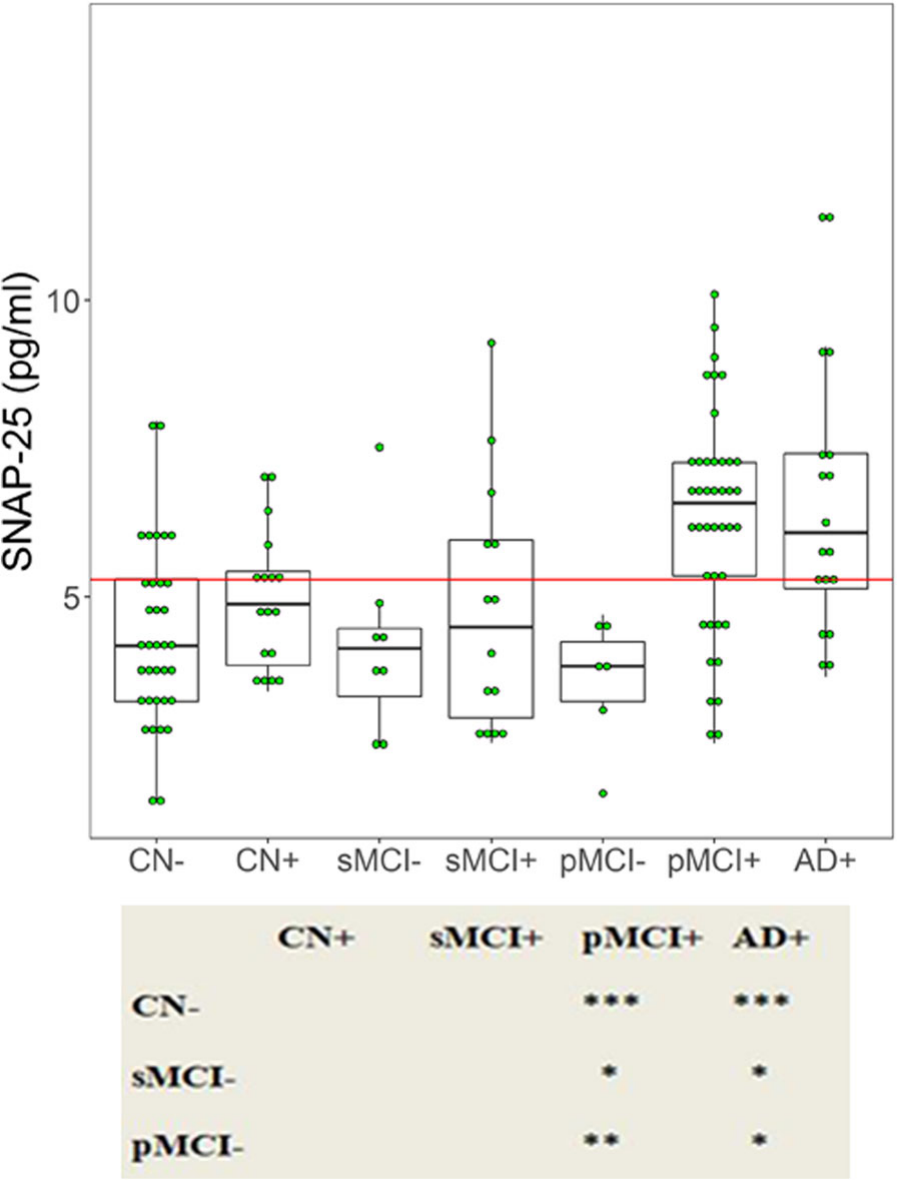
CSF SNAP-25 by diagnosis and amyloid pathology. The subjects included in the study were classified as Aβ^+^ or Aβ^–^. CSF SNAP-25 in different combinations of clinical diagnosis and Aβ pathology. Differences between groups were tested by multiple-variable linear regression, adjusted for age and sex. *p < 0.05, **p < 0.01, ***p < 0.0001. AD Alzheimer’s disease, CN cognitively normal, pMCI progressive mild cognitive impairment, sMCI stable mild cognitive impairment, SNAP-25 synaptosomal-associated protein 25

To evaluate the correlations between SNAP-25 and Aβ pathology, participants were dichotomized into CSF Aβ-positive (Aβ^+^) and Aβ-negative (Aβ^–^) using the previously established cutoff for CSF Aβ42 of 192 pg/ml [20]: CN Aβ^–^ (*n* = 35), CN Aβ^+^ (*n* = 17), sMCI Aβ^–^ (*n* = 8), sMCI Aβ^+^ (*n* = 14), pMCI Aβ^–^ (*n* = 6), pMCI Aβ^+^ (*n* = 41), and AD Aβ^+^ (*n* = 18). There were no participants with AD Aβ^–^ in this study. pMCI Aβ^+^ and AD Aβ^+^ patients had increased SNAP-25 levels compared with CN Aβ^–^ (*p* < 0.0001 for both groups) (Fig. 2). Furthermore, pMCI Aβ^+^ and AD Aβ^+^ cases had increased SNAP-25 levels compared with sMCI Aβ^–^ (*p* = 0.025 and *p* = 0.01, respectively) and pMCI Aβ^–^ (*p* = 0.004 and *p* = 0.036, respectively) subjects. sMCI Aβ^–^ and pMCI Aβ^–^ subjects had SNAP-25 levels in the same range as CN Aβ^–^ patients (Fig. 2).

**Fig. 3:**
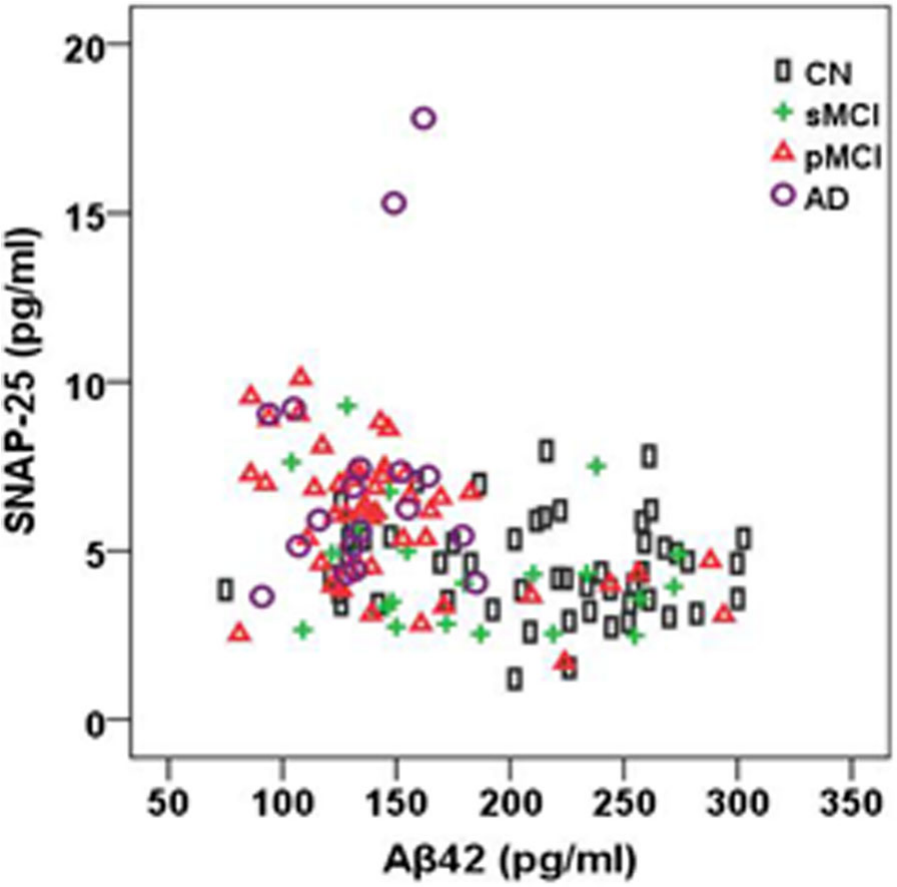
CSF SNAP-25 levels in relation to Aβ42. Correlations between CSF SNAP-25 levels and Aβ42 in different diagnostic groups. Aβ amyloid-β, AD Alzheimer’s disease, CN cognitively normal, pMCI progressive mild cognitive impairment, sMCI stable mild cognitive impairment, SNAP-25 synaptosomal-associated protein 25

There were no significant associations between SNAP-25 and Aβ42 in CN, sMCI, or AD subjects (*r* = −0.092, *p* = 0.518; *r* = −0.324, *p* = 0.142; *r* = 0.121, *p* = 0.633; respectively). SNAP-25 and Aβ42 were negatively correlated in pMCI patients (*r* = −0.389, *p* = 0.007) (Fig. 3). By nature of being a ratio with Aβ42, SNAP-25/Aβ42 will show a difference between Aβ42^+^ and Aβ42^–^ subjects. Therefore, we did not analyze the correlations between SNAP-25/Aβ42 ratio and Aβ pathology.

### Associations between CSF SNAP-25 and SNAP-25/Aβ42 and tau biomarkers

**Fig. 4:**
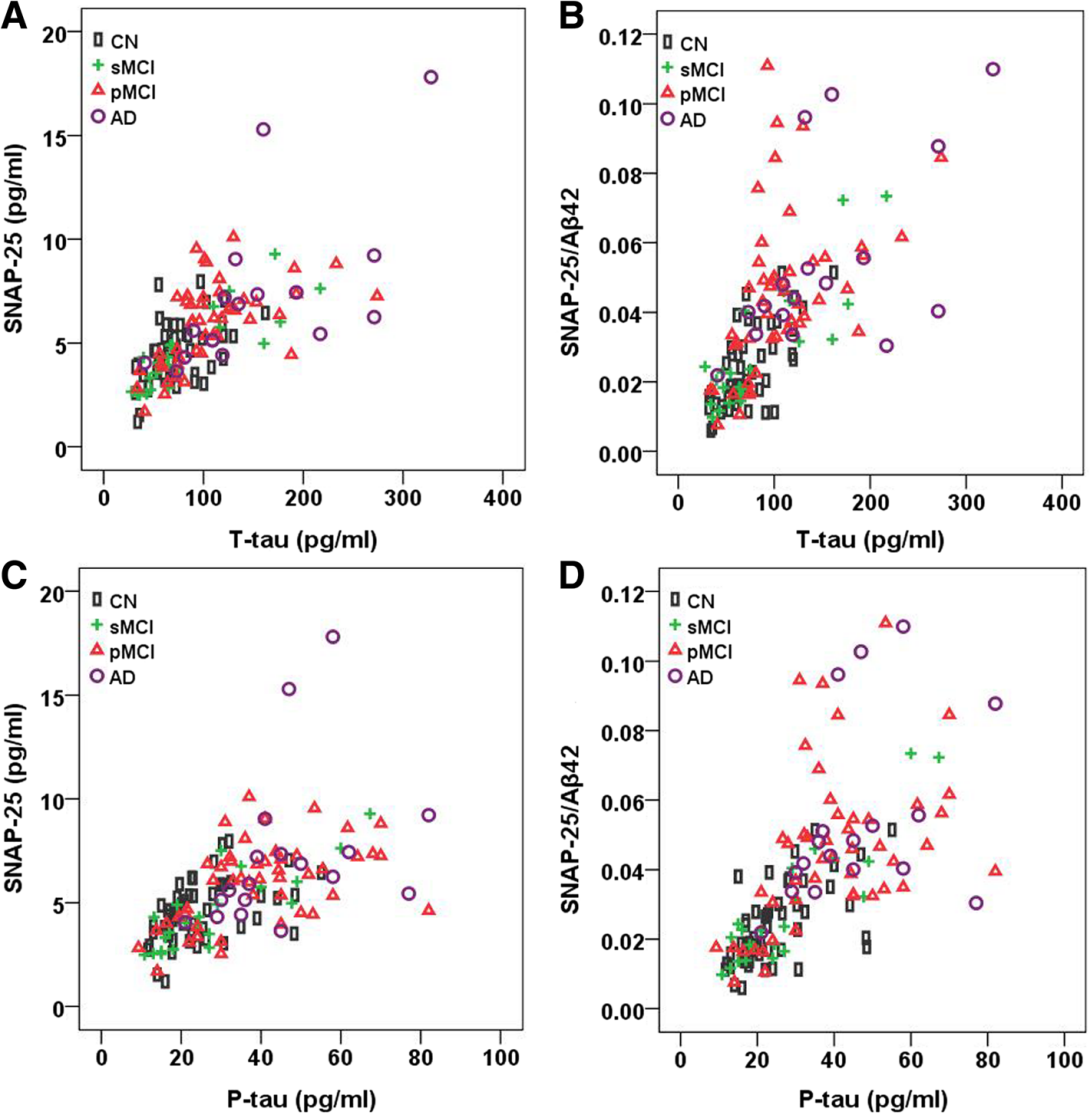
CSF SNAP-25 levels and SNAP-25/Aβ42 ratio in relation to tau biomarkers. Correlations between CSF SNAP-25 levels (**a**) and SNAP-25/Aβ42 ratio (**b**) and t-tau in different diagnostic groups. Correlations between CSF SNAP-25 levels (**c**) and SNAP-25/Aβ42 ratio (**d**) and p-tau in different diagnostic groups. Aβ amyloid-β, AD Alzheimer’s disease, CN cognitively normal, P-tau phosphorylated tau, pMCI progressive mild cognitive impairment, sMCI stable mild cognitive impairment, SNAP-25 synaptosomal-associated protein 25, T-tau total tau

t-tau and p-tau were strongly correlated with SNAP-25 in CN (*r* = 0.483, *p* < 0.001 for t-tau; *r* = 0.522, *p* < 0.001 for p-tau), sMCI (*r* = 0.876, *p* < 0.001 for t-tau; *r* = 0.858, *p* < 0.001 for p-tau), pMCI (*r* = 0.642, *p* < 0.001 for t-tau; *r* = 0.528, *p* < 0.001 for p-tau), and AD subjects (*r* = 0.791, *p* < 0.001 for t-tau; *r* = 0.644, *p* = 0.004 for p-tau) (Fig. 4a, c). t-tau and p-tau were also strongly correlated with SNAP-25/Aβ42 in all groups (Fig. 4b, d).

### Diagnostic accuracy of CSF SNAP-25, SNAP-25/Aβ42, and core CSF biomarkers

**Fig. 5:**
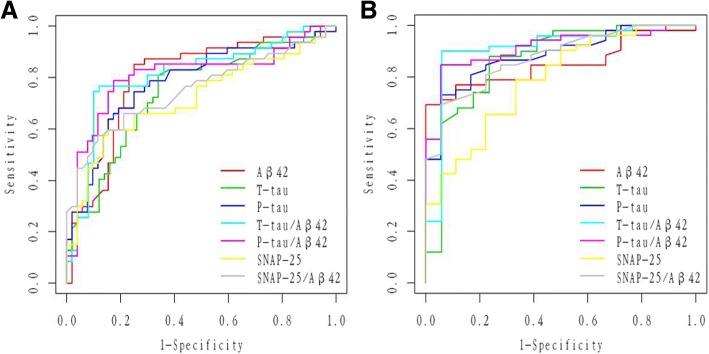
ROC analyses. ROC analyses were performed to test the CSF biomarkers and ratios in relation to clinical diagnoses for pMCI (**a**) and AD (**b**). Aβ amyloid-β, P-tau phosphorylated tau, SNAP-25 synaptosomal-associated protein 25, T-tau total tau

ROC analyses were performed to test CSF biomarkers and ratios in relation to clinical diagnoses for pMCI and AD. All CSF biomarkers and ratios had significant diagnostic accuracy for pMCI (Table 2 and Fig. 5a) and AD (Table 2 and Fig. 5b) compared with CN. Compared with Aβ42, t-tau, and p-tau, SNAP-25 had almost the same range of diagnostic accuracy for pMCI (SNAP-25 vs Aβ42, *p* = 0.21; SNAP-25 vs t-tau, *p* = 0.60; SNAP-25 vs p-tau, *p* = 0.18) (Table 2 and Fig. 5a) and AD (SNAP-25 vs Aβ42, *p* = 0.41; SNAP-25 vs t-tau, *p* = 0.11; SNAP-25 vs p-tau, *p* = 0.07) (Table 2 and Fig. 5b). Similarly, the SNAP-25/Aβ42 ratio appears to offer diagnostic accuracy at least as well as t-tau/Aβ42 and p-tau/Aβ42 for pMCI (SNAP-25/Aβ42 vs t-tau/Aβ42, *p* = 0.88; SNAP-25/Aβ42 vs p-tau/Aβ42, *p* = 0.93) (Table 2 and Fig. 5a) and AD (SNAP-25/Aβ42 vs t-tau/Aβ42, *p* = 0.79; SNAP-25/Aβ42 vs p-tau/Aβ42, *p* = 0.81) (Table 2 and Fig. 5b). In addition, SNAP-25/Aβ42 provided higher diagnostic accuracy than SNAP-25 alone for pMCI (*p* = 0.013) (Table 2 and Fig. 5a) and AD (*p* = 0.015) (Table 2 and Fig. 5b). However, t-tau/Aβ42 and p-tau/Aβ42 ratios provided higher diagnostic accuracy than SNAP-25 alone for AD (*p* = 0.044 and *p* = 0.038, respectively) (Table 2 and Fig. 5b) but not pMCI (*p* = 0.06 for both) (Table 2 and Fig. 5a).

**Table 2:** AUC of CSF biomarkers

	SNAP-25	Aβ42	t-tau	p-tau	SNAP-25/Aβ42	t-tau/Aβ42	p-tau/Aβ42
pMCI	0.72 (0.61–0.82) (*p* < 0.001)	0.80 (0.70–0.89) (*p* < 0.001)	0.74 (0.65–0.84) (*p* < 0.001)	0.78 (0.69–0.88) (*p* < 0.001)	0.81 (0.72–0.90) (*p* < 0.001)	0.81 (0.72–0.90) (*p* < 0.001)	0.81 (0.72–0.90) (*p* < 0.001)
AD	0.79 (0.67–0.91) (*p* = 0.002)	0.85 (0.77–0.94) (*p* < 0.001)	0.87 (0.75–0.98) (*p* < 0.001)	0.88 (0.80–0.96) (*p* < 0.001)	0.91 (0.84–0.98) (*p* < 0.001)	0.91 (0.82–1.00) (*p* < 0.001)	0.91 (0.84–0.98) (*p* < 0.001)

*Aβ* amyloid-β, *AD* Alzheimer’s disease, *AUC* area under the receiver operator characteristics curve, *CSF* cerebrospinal fluid, *pMCI* progressive mild cognitive impairment, *p-tau* phosphorylated tau, *SNAP-25* synaptosomal-associated protein 25, *t-tau* total tau

### CSF SNAP-25 and SNAP-25/Aβ42 predict conversion from MCI to AD

**Fig. 6:**
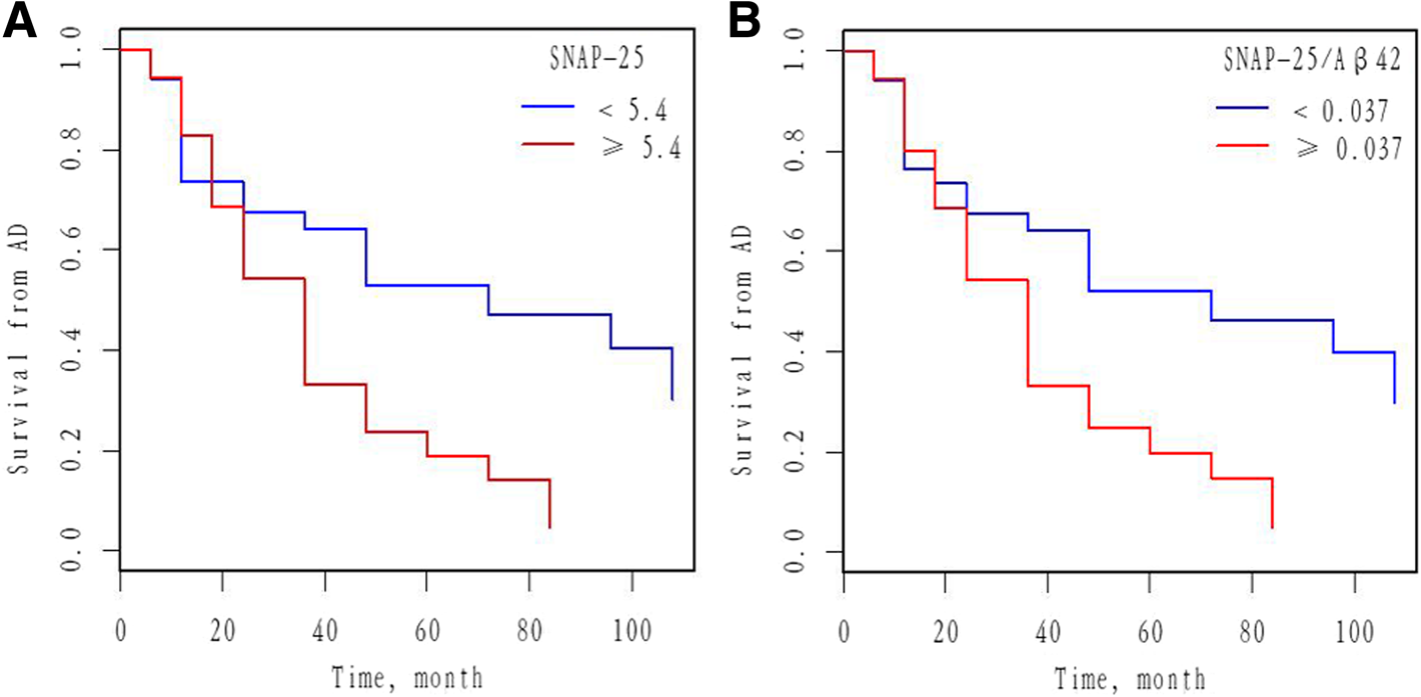
Baseline CSF measures of SNAP-25 and SNAP-25/Aβ42 as predictors of conversion from MCI to AD. Survival from AD as a function of CSF SNAP-25 (**a**) and SNAP-25/Aβ42 (**b**) measures (dichotomized at the median values) are shown. Analyses were adjusted for age and gender. Cutoff values were 5.4 pg/ml and 0.037 for SNAP-25 and SNAP-25/Aβ42, respectively. Aβ amyloid-β, AD Alzheimer’s disease, SNAP-25 synaptosomal-associated protein 25

We investigated whether CSF SNAP-25 and SNAP-25/Aβ42 predicted conversion from MCI to AD. Cox proportional hazard models were performed for SNAP-25 and SNAP-25/Aβ42 as a continuous variable after adjusting for age and gender. CSF SNAP-25 and SNAP-25/Aβ42 significantly predicted conversion from MCI to AD. HRs were then calculated for SNAP-25 and SNAP-25/Aβ42 as a dichotomous variable using the median values of SNAP-25 and SNAP-25/Aβ42 as a cutoff (adjusting for age and gender). Individuals with high SNAP-25 (HR 2.47, *p* = 0.011), corresponding to individuals whose SNAP-25 values were ≥ 5.4 pg/ml, progressed much more rapidly to AD than individuals with lower values (< 5.4 pg/ml, corresponding to the lower median values of SNAP-25) (Fig. 6a). Individuals with high SNAP-25/Aβ42 (HR 2.41, *p* = 0.013), corresponding to individuals whose SNAP-25/Aβ42 values were ≥ 0.037, progressed much more rapidly to AD than individuals with lower values (< 0.037, corresponding to the lower median values of SNAP-25/Aβ42) (Fig. 6b).

### CSF SNAP-25 and SNAP-25/Aβ42 in relation to cognition

**Fig. 7:**
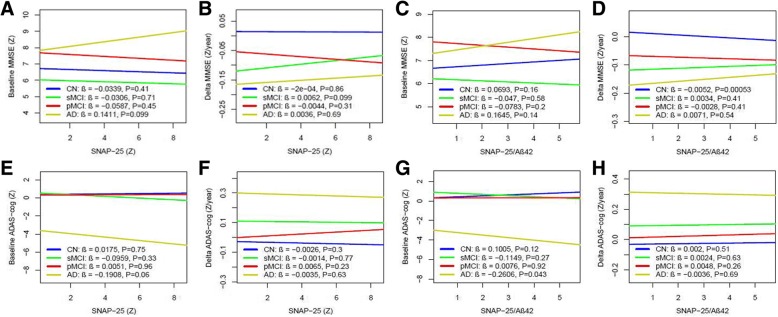
CSF SNAP-25 and SNAP-25/Aβ42 in relation to cognition and future cognitive change. MMSE and ADAS-cog at baseline (**a, e**) and over time (**b, f**) as a function of baseline CSF SNAP-25 in different diagnostic groups. MMSE and ADAS-cog at baseline (**c, g**) and over time (**d, h**) as a function of baseline SNAP-25/Aβ42 in different diagnostic groups. Biomarker levels and ratios are standardized to *z* scores. Aβ amyloid-β, AD Alzheimer’s disease, ADAS-cog Alzheimer’s Disease Assessment Scale cognitive subscale, CN cognitively normal, MMSE Mini-Mental State Examination, pMCI progressive mild cognitive impairment, sMCI stable mild cognitive impairment, SNAP-25 synaptosomal-associated protein 25

CSF SNAP-25 did not correlate with MMSE or ADAS-cog scores at baseline or rates of change of MMSE and ADAS-cog during follow-up (Fig. 7a, b, e, f). In contrast, SNAP-25/Aβ42 ratio correlated with ADAS-cog scores in AD at baseline (β = −0.26, *p* = 0.043) (Fig. 7g) and a decreased changing rate of MMSE for CN during the clinical follow-up period (β = −0.01, *p* < 0.001) (Fig. 7d).

### CSF SNAP-25 and SNAP-25/Aβ42 in relation to brain structure and WMH

**Fig. 8:**
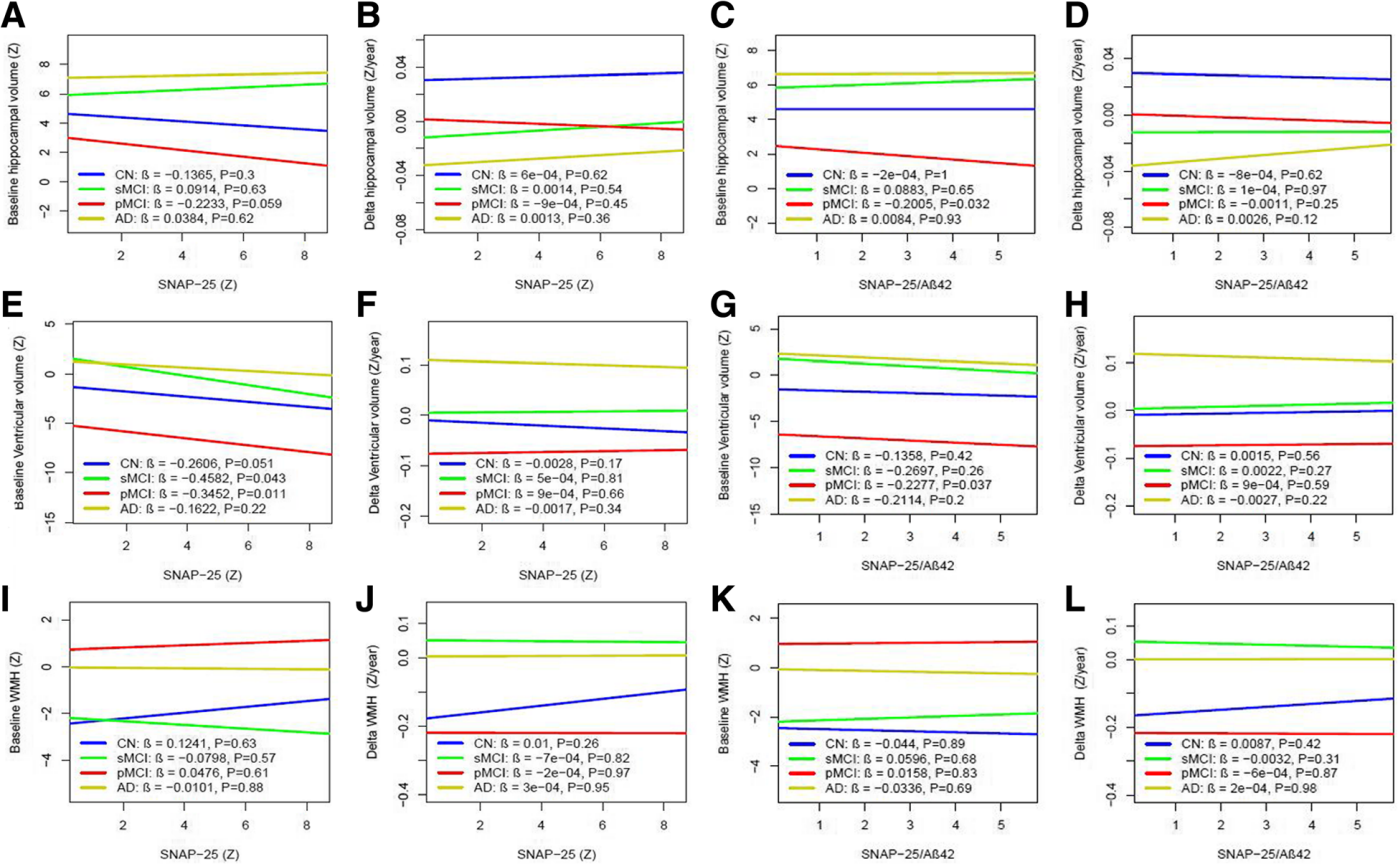
CSF SNAP-25 and SNAP-25/Aβ42 in relation to brain structure and WMH. Hippocampal volume, ventricular volume, and WMH at baseline (**a**, **e**, **i**) and over time (**b**, **f**, **j**) as a function of baseline CSF SNAP-25 in different diagnostic groups. Hippocampal volume, ventricular volume, and WMH at baseline (**c**, **g**, **k**) and over time (**d**, **h**, **l**) as a function of baseline SNAP-25/Aβ42 in different diagnostic groups. Biomarker levels and ratios are standardized to *z* scores. Aβ amyloid-β, AD Alzheimer’s disease, CN cognitively normal, pMCI progressive mild cognitive impairment, sMCI stable mild cognitive impairment, SNAP-25 synaptosomal-associated protein 25, WMH white matter hyperintensity

Finally, we examined whether SNAP-25 and SNAP-25/Aβ42 correlated with hippocampal volumes, ventricular volumes, and WMH as measured with MRI (Fig. 8). SNAP-25 was associated with baseline smaller ventricular volumes in sMCI (β = −0.46, *p* = 0.043) and pMCI (β = −0.35, *p* = 0.011) groups (Fig. 8e), but was not associated with baseline hippocampal volumes or WMH in any group (Fig. 8a, i). SNAP-25 was not associated with rates of change of brain structure or WMH during the follow-up period in any group (Fig. 8b, i, j). SNAP-25/Aβ42 ratio was associated with baseline hippocampal (β = −0.20, *p* = 0.032) (Fig. 8c) and smaller ventricular volumes (β = −0.23, *p* = 0.037) (Fig. 8g) in the pMCI group.

## Discussion

The present study investigated the relationships between SNAP-25, a biomarker of synaptic loss, and other key biomarkers across the AD spectrum. The results demonstrate that SNAP-25 markers increase with disease severity and are able to distinguish between diagnostic groups. Finally, markers of synapse loss were predictive of clinical progression to dementia and neurodegeneration.

For many years, the classic definition of neurodegenerative diseases such as AD was limited to the discovery of selective neuronal loss and astrogliosis. This concept, however, has now been extended to include neuroinflammation and synaptic loss [9]. Synaptic loss precedes neuronal loss and correlates with early deficits in memory formation [22]. SNAP-25 is a characteristic component of synapses and is highly expressed in the central nervous system. The absence of SNAP-25 may not stop neurotransmission, but it is associated with several physiological functions including synaptic vesicle release and recycling, neurite extension, neuron repair, and synaptogenesis [23, 24]. SNAP-25 is involved in the regulation of long-term potentiation and formation of long-term memory in the hippocampus CA3 region, which is consistent with its role in learning and memory functions in the hippocampal CA1 area [25]. Since previous studies have shown that SNAP-25 was detectable in CSF [26], this presynaptic protein can be used as a biomarker to monitor the molecular pathogenesis of AD that was previously difficult to assess in living patients.

CSF SNAP-25 was significantly increased in pMCI and AD compared with the cognitively normal. In the present study, we also show that pMCI patients had increased CSF SNAP-25 levels compared with sMCI individuals. SNAP-25/Aβ42 ratio had the same trend in every diagnostic group. Interestingly, 19 cognitively normal subjects who progressed to MCI or AD during follow-up had increased SNAP-25/Aβ42 ratio (but not SNAP-25) compared with the other cognitively normal subjects. This finding suggests that CSF SNAP-25, especially the SNAP-25/Aβ42 ratio, is an early pathophysiological indicator of AD-related synaptic loss.

To investigate whether CSF SNAP-25 levels correlated with underlying Aβ pathology, each group included in the study was dichotomized into Aβ^+^ and Aβ^–^. As expected, the CSF SNAP-25 levels were significantly increased in the CSF AD Aβ^+^ group. Similarly, high CSF SNAP-25 levels were also found in the pMCI Aβ^+^ group. In addition, SNAP-25 and Aβ42 were negatively correlated in pMCI patients. Currently, the general view is that AD progresses from CN Aβ^–^ to CN Aβ^+^, MCI Aβ^+^, and finally AD Aβ^+^, while non-Aβ-dependent cognitive decline may progress from CN Aβ^–^ to MCI Aβ^–^ and AD Aβ^–^ [27]. In addition, t-tau and p-tau were strongly correlated with SNAP-25 and SNAP-25/Aβ42 ratio in every diagnostic group, suggesting that SNAP-25 [28] or SNAP-25/Aβ42 ratio may be a surrogate biomarker for future clinical treatment studies with tau-modifying drugs. Interestingly, we observed the strongest correlation between SNAP-25 and tau in sMCI patients. We do not know the specific reason for this, but we speculate that tau protein pathology plays a more important role in the pathogenesis of sMCI.

In the present study, our results show that CSF SNAP-25, especially SNAP-25/Aβ42, offers diagnostic sensitivity for AD that is comparable with that of CSF Aβ42, t-tau, p-tau, t-tau/Aβ42, and p-tau/Aβ42. CSF SNAP-25 and SNAP-25/Aβ42 also have high diagnostic sensitivity for pMCI. In addition, SNAP-25/Aβ42 appears to offer diagnostic sensitivity at least as well as the current “gold standard” prognostic biomarkers for AD, such as t-tau/Aβ42 and p-tau/Aβ42. These findings, therefore, suggest CSF SNAP-25 and SNAP-25/Aβ42 ratio as diagnostic biomarkers for the earliest symptomatic stage of AD.

There is accumulating evidence that synaptic loss is a surrogate for disease progression in AD [7]. To the best of our knowledge, no studies have evaluated the predictive value of SNAP-25 for the conversion from MCI to AD. Here, we demonstrate that CSF SNAP-25 and SNAP-25/Aβ42 offer predictive value for future disease progression in MCI subjects. Our findings suggest that CSF SNAP-25 and SNAP-25/Aβ42 may complement the prognostic utility of CSF Aβ42, t-tau, and p-tau in predicting the evolution of cognitive impairment.

Evidence suggests that progressive neuronal and synaptic loss are the surrogate markers for cognitive deterioration in AD [7]. In the present study, although CSF SNAP-25 does not correlate with cognitive decline at baseline and follow-up, our results suggest that the SNAP-25/Aβ42 ratio may offer predictive value for future cognitive impairment in cognitively normal subjects. In our study, 19 cognitively normal subjects who progressed to MCI or AD during follow-up had increased SNAP-25/Aβ42 ratio compared with stably cognitive normal subjects, which implies that the combination of SNAP-25 and Aβ42 (SNAP-25/Aβ42) might be useful to follow progression of cognitive decline. However, SNAP-25 levels were similar between stably cognitive normal subjects and progressively cognitive normal subjects, and Aβ42 levels in progressively cognitive normal subjects were less than those in stably cognitive normal subjects. Thus, these results do not suggest that SNAP-25 has added predictive value on top of Aβ42 alone for cognitively normal subjects. We also found associations between high SNAP-25/Aβ42 ratio and an increased rate of hippocampal atrophy. Given that synaptic dysfunction and loss are probably the major causes of the loss of neuropil underlying hippocampal atrophy [12], we believe that SNAP-25/Aβ42 may be an independent novel biomarker for synaptic pathology in AD, and the clinical manifestations of cognitive impairment and later dementia appear after neuronal injury and synaptic loss has reached a threshold in vulnerable brain regions. This study has limitations. First, our cases do not include non-AD neurodegenerative diseases, and our study is not a pathological study; thus it lacks pathological evidence. Second, the ADNI database was volunteered by highly educated individuals for research focused on AD research. This may give rise to bias in choice because the study population is a self-selected individual who may have concerns about their cognition. Third, the convenience samples used in this study may limit the universality of our findings. Finally, the sample size of this study is relatively small. Therefore, it is necessary to replicate our results in larger population-based cohorts and to conduct research on the related mechanisms.

## Conclusions

In summary, CSF SNAP-25, especially the SNAP-25/Aβ42 ratio, was already increased in the predementia stages of AD, and higher concentrations correlate with a higher rate of cognitive decline and hippocampal atrophy at some stages of AD. These findings may highlight the potential use of CSF SNAP-25 and SNAP-25/Aβ42 in trial designs, in response to therapies in clinical trials of disease-modifying therapies, in treatment decisions, and outcome assessments, and may complement diagnostic and prognostic information provided by CSF Aβ42, t-tau, and p-tau.

## Abbreviations

Aβ: Amyloid-β
AD: Alzheimer’s disease
ADAS-cog: Alzheimer’s Disease Assessment Scale cognitive subscale
ADNI: Alzheimer’s disease Neuroimaging Initiative
ANOVA: Analysis of covariance
AUC: Area under the curve
CDR: Clinical Dementia Rating
CI: Confidence interval
CN: Cognitively normal
CSF: Cerebrospinal fluid
HR: Hazard ratio
MCI: Mild cognitive impairment
MMSE: Mini-Mental State Examination
MRI: Magnetic resonance imaging
pCN: Progressively cognitive normal
PET: Positron emission tomography
pMCI: Progressive mild cognitive impairment
p-tau: Phosphorylated tau
ROC: Receiver operating curve
sCN: Stably cognitive normal
sMCI: Stable mild cognitive impairment
SNAP-25: Synaptosomal-associated protein 25
t-tau: Total tau
WMH: White matter hyperintensity

## References

[1] DeKosky ST, Scheff SW, Styren SD (1996). Structural correlates of cognition in dementia: quantification and assessment of synapse change. Neurodegeneration.

[2] Terry RD, Masliah E, Salmon DP, Butters N, DeTeresa R, Hill R, Hansen LA, Katzman R (1991). Physical basis of cognitive alterations in Alzheimer’s disease: synapse loss is the major correlate of cognitive impairment. Ann Neurol.

[3] DeKosky ST, Scheff SW (1990). Synapse loss in frontal cortex biopsies in Alzheimer’s disease: correlation with cognitive severity. Ann Neurol.

[4] Furuya TK, Silva PN, Payao SL, Bertolucci PH, Rasmussen LT, De Labio RW, Braga IL, Chen ES, Turecki G, Mechawar N, Mill J, Smith MA (2012). Analysis of SNAP25 mRNA expression and promoter DNA methylation in brain areas of Alzheimer's disease patients. Neuroscience.

[5] Lista S, Hampel H (2017). Synaptic degeneration and neurogranin in the pathophysiology of Alzheimer’s disease. Expert Rev Neurother.

[6] Scheff SW, DeKosky ST, Price DA (1990). Quantitative assessment of cortical synaptic density in Alzheimer’s disease. Neurobiol Aging.

[7] Ingelsson M, Fukumoto H, Newell KL, Growdon JH, Hedley-Whyte ET, Frosch MP, Albert MS, Hyman BT, Irizarry MC (2004). Early Abeta accumulation and progressive synaptic loss, gliosis, and tangle formation in AD brain. Neurology.

[8] Scheff SW, Sparks L, Price DA (1993). Quantitative assessment of synaptic density in the entorhinal cortex in Alzheimer’s disease. Ann Neurol.

[9] Overk CR, Masliah E (2014). Pathogenesis of synaptic degeneration in Alzheimer’s disease and Lewy body disease. Biochem Pharmacol.

[10] Counts SE, Alldred MJ, Che S, Ginsberg SD, Mufson EJ (2014). Synaptic gene dysregulation within hippocampal CA1 pyramidal neurons in mild cognitive impairment. Neuropharmacology.

[11] Scheff SW, Price DA, Schmitt FA, DeKosky ST, Mufson EJ (2007). Synaptic alterations in CA1 in mild Alzheimer disease and mild cognitive impairment. Neurology.

[12] Portelius E, Zetterberg H, Skillback T, Tornqvist U, Andreasson U, Trojanowski JQ, Weiner MW, Shaw LM, Mattsson N, Blennow K (2015). Cerebrospinal fluid neurogranin: relation to cognition and neurodegeneration in Alzheimer’s disease. Brain.

[13] Pham E, Crews L, Ubhi K, Hansen L, Adame A, Cartier A, Salmon D, Galasko D, Michael S, Savas JN, Yates JR, Glabe C, Masliah E (2010). Progressive accumulation of amyloid-beta oligomers in Alzheimer’s disease and in amyloid precursor protein transgenic mice is accompanied by selective alterations in synaptic scaffold proteins. FEBS J.

[14] Greber S, Lubec G, Cairns N, Fountoulakis M (1999). Decreased levels of synaptosomal associated protein 25 in the brain of patients with Down syndrome and Alzheimer’s disease. Electrophoresis.

[15] Brinkmalm A, Brinkmalm G, Honer WG, Frolich L, Hausner L, Minthon L, Hansson O, Wallin A, Zetterberg H, Blennow K, Ohrfelt A (2014). SNAP-25 is a promising novel cerebrospinal fluid biomarker for synapse degeneration in Alzheimer’s disease. Mol Neurodegener.

[16] Berg L (1988). Clinical dementia rating (CDR). Psychopharmacol Bull.

[17] Folstein MF, Folstein SE, McHugh PR (1975). “Mini-mental state”. A practical method for grading the cognitive state of patients for the clinician. J Psychiatr Res.

[18] Aisen PS, Petersen RC, Donohue MC, Gamst A, Raman R, Thomas RG, Walter S, Trojanowski JQ, Shaw LM, Beckett LA, Jack CR, Jagust W, Toga AW, Saykin AJ, Morris JC, Green RC, Weiner MW (2010). Clinical core of the Alzheimer’s disease neuroimaging initiative: progress and plans. Alzheimers Dement.

[19] Tierney MC, Fisher RH, Lewis AJ, Zorzitto ML, Snow WG, Reid DW, Nieuwstraten P (1988). The NINCDS-ADRDA work group criteria for the clinical diagnosis of probable Alzheimer’s disease: a clinicopathologic study of 57 cases. Neurology.

[20] Shaw LM, Vanderstichele H, Knapik-Czajka M, Clark CM, Aisen PS, Petersen RC, Blennow K, Soares H, Simon A, Lewczuk P, Dean R, Siemers E, Potter W, Lee VM, Trojanowski JQ (2009). Cerebrospinal fluid biomarker signature in Alzheimer’s disease neuroimaging initiative subjects. Ann Neurol.

[21] Risacher SL, Saykin AJ (2013). Neuroimaging and other biomarkers for Alzheimer’s disease: the changing landscape of early detection. Annu Rev Clin Psychol.

[22] Arendt T (2009). Synaptic degeneration in Alzheimer’s disease. Acta Neuropathol.

[23] Bark IC, Hahn KM, Ryabinin AE, Wilson MC (1995). Differential expression of SNAP-25 protein isoforms during divergent vesicle fusion events of neural development. Proc Natl Acad Sci U S A.

[24] Walch-Solimena C, Blasi J, Edelmann L, Chapman ER, von Mollard GF, Jahn R (1995). The t-SNAREs syntaxin 1 and SNAP-25 are present on organelles that participate in synaptic vesicle recycling. J Cell Biol.

[25] Hou QL, Gao X, Lu Q, Zhang XH, Tu YY, Jin ML, Zhao GP, Yu L, Jing NH, Li BM (2006). SNAP-25 in hippocampal CA3 region is required for long-term memory formation. Biochem Biophys Res Commun.

[26] Davidsson P, Puchades M, Blennow K (1999). Identification of synaptic vesicle, pre- and postsynaptic proteins in human cerebrospinal fluid using liquid-phase isoelectric focusing. Electrophoresis.

[27] Mattsson N, Insel PS, Palmqvist S, Portelius E, Zetterberg H, Weiner M, Blennow K, Hansson O (2016). Cerebrospinal fluid tau, neurogranin, and neurofilament light in Alzheimer’s disease. EMBO Mol Med.

[28] Salomone S, Caraci F, Leggio GM, Fedotova J, Drago F (2012). New pharmacological strategies for treatment of Alzheimer’s disease: focus on disease modifying drugs. Br J Clin Pharmacol.

